# Implementation of a Sustainable Training System for Emergency in Vietnam

**DOI:** 10.3389/fpubh.2018.00004

**Published:** 2018-01-29

**Authors:** Sunjoo Kang, Hyejin Seo, Binh Duy Ho, Phuong Thi Anh Nguyen

**Affiliations:** ^1^Department of Nursing, Cheju Halla University, Jeju City, South Korea; ^2^Halla-Stony Brook Emergency Medicine Education Center, Cheju Halla University, Jeju City, South Korea; ^3^Hue University of Medicine and Pharmacy, Hue, Vietnam

**Keywords:** sustainable training, emergency medicine, medical education, project evaluation, Vietnam

## Abstract

**Purpose:**

This study analyzed the project outcomes to share lessons regarding the development of an emergency medicine education system in Vietnam.

**Methods:**

Retrospective evaluation was implemented using project outcome indicators.

**Results:**

A total of 13 training courses were administered, with the collaboration of international experts in Korea and Vietnam. A total of 23 kinds of emergency medicine education equipment were purchased, and a basic life support (BLS) and two advanced cardiac life support labs were remodeled to provide appropriate simulation training. Throughout the 2 years of the project, nine Vietnamese BLS instructors were approved by the Korea Association of Cardiopulmonary Resuscitation under American Heart Association. Results of evaluation by Korean international development experts were based on five criteria, provided by the Development Assistance Committee of the Organization for Economic Co-operation and Development, were excellent. Success factors were identified as partnership, ownership, commitment, government support, and global networking.

**Conclusion:**

Project indicators were all accomplished and received an excellent evaluation by external experts. For sustainable success, healthcare policy and legal regulation to promote high quality and safe service to the Vietnamese people are recommended.

## Introduction

Strengthening health systems can promote a healthy population and result in national development, according to the World Bank (1). Formerly, the World Health Organization (WHO) recommended six building blocks for a national healthcare system and published several monitoring and capacity building strategies for each factor (2, 3). However, in a low-resource setting, often the last mandate developed in healthcare is emergency medicine, which, while contributing to saving lives in the pre- or in-hospital phase of life-threatening situations, is an enormously demanding financial burden (4). Interestingly, several studies have reported that emergency medicine and its training/education is not as quite expensive as previously thought, and is likely to be more dependent on how problems are solved (5–7). Therefore, a realistic and non-demanding financial burden approach should be considered for the fundamental capacity building of the emergency medicine field in underfunded countries.

There is evidence of a global change in disease patterns, with low-, middle-, and high-income countries exhibiting similar trends and direction regarding the national burden of disease (8). The global survival rate from non-communicable disease is approximately 70%. However, this number increased to over 88% in high-income countries and decreased to 37% in low-income ones (9). This implies that the global burden of disease, which is calculated by summing the disease burden of individual nations, does not accurately reflect that the majority of global disease burden is related to economic status. For the past 30 years, the annual income and life expectancy in Vietnam have increased remarkably, from about $200 to $1,900 and from 68.5 to 75.6 years (10), respectively. Conversely, in the same duration, life expectancy increased from 67.9 to 82.2 years, and annual income from $2,350 to $26,970 in the Republic of Korea (11) Vietnam’s economic development and the potential capacity of its people have fostered optimism for the future prospects of the country throughout the region.

The Korean government has cooperated with developing countries, especially focusing on the Asia-Pacific region. Since 2012, the National Research Foundation (NRF) of Korea, under the Ministry of Education, has implemented official development assistance, supporting higher educational institutions in Korea to propose 4-year projects that can transfer knowledge to institutions in partner countries. The new NRF project was slated for 4 years and divided into two phases. The project development and implementation process, from situational analysis to evaluation, is shown. The first phase of the project was operated from June 2014 to May 2016. Sub-objectives of the first phase were to train basic life support (BLS) instructors appropriately, receive the approval as an international training organization from the American Heart Association (AHA), renovate advanced emergency medicine education system environment, provide training program for healthcare providers by Vietnamese instructors, accomplish AHA approval criteria, and make further efforts to raise the baseline of human capacity, especially through simulation as a training method and equipment operation. Project activities were totally dependent on the request of the Vietnam partner, as the role of the Korean project team was to mentor, and empower the Vietnam partner, resulting in a feeling of ownership, and the ability to successfully manage and sustain the implemented changes following project cessation. There was a consensus to build the capacity of healthcare personnel supplement program operations, especially to meet AHA approval and develop a more relevant program with an appropriate environment to provide training. The goal of the second phase was to expand community emergency capacity through first aid training.

This study analyzed the outcomes to share the lessons learned from the first phase of project for construction of emergency medicine education system in Vietnam.

## Background and Rationale

### Background

In 2013, the project team first visited Hue University of Medicine and Pharmacy (Hue UMP), Vietnam, when one of the Korean project team was implementing a project in Vietnam. This past project focused on the capacity building of nurses in seven hospitals in Binh Dinh province, Vietnam (12). At the beginning of the previous project, the level of autonomy and empowerment of Vietnam nurse educators were not activated. In response to this, one of the Korean project teams visited the Hue UMP nursing faculty to discuss how to implement nurse training programs that were appropriate for the organizational culture of Vietnam hospitals (13). With the collaboration of local experts from the faculty of nursing, Hue UMP, the project team designed a 1-week long program to train facilitators. This 1-week training was held at Hue UMP using their infrastructure of one classroom and two practice labs, and including four Vietnamese instructors and two Korean instructors, at the end of February 2014. The experience of the previous project helped in developing an understanding of the partner institution’s potential needs to improve their education system (12). However, there was also a serious lack of an appropriate environment and standardized program, especially in emergency medicine supplementary trainings, such as BLS, advanced cardiopulmonary life support (ACLS), basic trauma life support, advanced trauma life support, airway management, disaster management, and/or other critical care skills at Hue UMP. Most former training done in Hue UMP was dependent on international specialists or a non-governmental organization (i.e., the GSMM), with no self-operated, well-organized programs present (14).

### Rationale

Vietnam’s socioeconomic development appears to be similar to that experienced by the Republic of Korea, 40 years ago (15). Vietnam’s healthcare system has a public sector dominant delivery system, with only 4% of the total beds owned by private hospitals. The public sector consists of central and specialized hospitals, provincial and district hospitals, community health stations, and village health workers. Conversely, the private sector is composed of drug vendors, general practitioners, private pharmacies, and nursing homes (16). This means that governmental regulations and policies can exert a powerful effect on health, and can do so more effectively than any other country with a dominant private health sector. Like many other developing countries, it did not take long to develop a full understanding of the emergency medicine field in Vietnam (4, 6). The emergency medicine system was not activated in Vietnam until 2009. The activation of this system was accelerated by the First International Symposium in Emergency Medicine held in Hue in March 2010, which was a significant landmark in the history of emergency medicine in Vietnam (17, 18).

Hue UMP was the first institution to begin a residency program for emergency medicine in Vietnam. Additionally, the Vietnam Society of Emergency Medicine was organized in 2013; the Vietnam Emergency Medicine Development Project, in collaboration with the Good Samaritan Medical Ministry (GSMM), provided both an emergency medicine exchange teaching program and an annual emergency medicine conference (14). Further, staff from Hue province 115 center, which is the community-based center that dispatches ambulances to patients, evacuates patients to an emergency center, and provides emergency first-aid treatment during transport (similar to 911 in America or 119 in Korea), visited the Republic of Korea to benchmark the system in 2005. During this visit, 115 center staffed doctors, nurses, pharmacists, and some administration, but not a paramedic. The 115 centers in Hue province had their own in-service program; however, a more systematic program was identified as necessary through interviews (19). This center also had its own defibrillator and ventilator, but these were operated very little each month, only when a serious patient call was received. Moreover, public automated electric defibrillators were not in service.

Overall, the fundamental infrastructure of the emergency medical education system, addressing care between pre-hospital and in-hospital, was not well organized (12). As changes in patterns of disease in Vietnam have shown a similar trajectory to developed countries, it was imperative to develop a standard emergency first-aid program for both healthcare personnel and community members (15).

## Methods

### Design

This study is a retrospective project evaluation of construction of an emergency medicine education system through official development assistance from Vietnam and South Korea.

### Research Subjects

Nine candidates for the role of BLS instructor among faculty of nursing, Hue UMP were the main subjects for instructor training in this project. Two of them were nurses and the others were medical doctors. All study participants provided their verbal informed consent before participating in the initial course.

### Data Collection

Data were collected to monitor project outcome indicators through the project process and these reported on each activity. Ethical approval of using the data obtained was not required according to the “Memorandum of Project Implementation.”

### Data Analysis

The collected data were reviewed using project indicators. The frequency and percentage of the subjects’ pass rates were analyzed.

### Limitation of the Study

This research was conducted with a 2-year experience out of the 4-year project, and so caution should be taken in generalizing the results.

## Results

### Human Resource Development for BLS Instructors

The AHA’s criteria for the approval of an international training organization are extremely standardized. The necessary activity for this approval was to train BLS instructors in an AHA approved method. Programs to train Vietnamese BLS instructors included four sequential trainings (provider, pre-monitor, instructor, and post-monitor courses). The BLS provider course was opened at three time points in October and also in December 2014 in Korea at Halla-Stony Emergency Medicine Education Center (Halla-Stony EMEC) in Cheju Halla University, which is the official BLS training site of KACPR and AHA. Any BLS participant who passed the written and skill tests was deemed as an appropriate instructor candidate. The pre-monitor course was then offered to these individuals. In the first year, only four of nine participants passed the BLS provider course with a high score. Pre-monitor, instructor, and post-monitor courses were held in December 2014 for these four candidates in Korea, and the Vietnamese BLS provider candidates were the subject of an instructor course and post-monitor course, as shown in Tables 1 and 2. Another instructor course was initiated for five candidates who failed the first provider course, but passed a second provider course in October 2015. At this time, an American BLS instructor taught together with Korean instructors.

**Table 1:** Courses for training Vietnamese basic life support (BLS) instructors.

When	Trained by	No.	Where	Course	Duration	Trainees
October 2014	Korean BLS faculty	2	South Korea	American Heart Association (AHA) BLS provider	1 day	8
	American BLS instructor	2				
	American instructor	2	South Korea	Difficult airway	2 days	8
	Korean BLS instructor	3				

December 2014	Korean BLS faculty	2	South Korea	AHA BLS provider	1 day	4
	Korean faculty	2		AHA BLS pre-monitor	1 day	4
	Korean faculty	2		AHA BLS instructor	1 day	4
	Korean BLS instructor	1		AHA BLS instructor	1 day	4
	Korean instructor	1		AHA BLS post-monitor	1 day	4

February 2015	Korean BLS faculty	1	Vietnam	Simulation on training equipment	2× 3 h	All participants
	Korean BLS instructor	1	Vietnam	Pilot BLS course for medical and nursing students	2× 6 h	24 students/course

October 2015	Korean BLS faculty	4	South Korea	AHA BLS provider	1 day	2
	Korean faculty	4		AHA BLS pre-monitor	1 day	5
	American BLS Instructor	1		pre-monitor	1 day	5
	American instructor	1		AHA BLS instructor	1 day	5
	Korean BLS instructor	3		AHA BLS instructor	1 day	5
	Korean instructor	3		AHA BLS post-monitor	1 day	5

February 2016	Korean BLS faculty	1	Vietnam	Simulation on course operation and equipment	2× 3 h	All participants
	Korean BLS instructor	1		Pilot BLS course for medical and nursing students	2× 6 h	24 students/course

**Table 2:** Basic life support (BLS) and difficult airway course schedule.

Course: BLS (provider, instructor, pre-, and post-monitor)		

Time	Content	
08:20–08:30	Registration	
08:30–09:00	Pretest review	
09:00–09:10	Course introduction	
09:10–09:30	2010 American Heart Association (AHA) Guidelines	
09:30–10:20	BLS/CPR basic for adults – Chest compression – Breaths with pocket-mask and bag-mask for adult victims – 1-rescuer adult BLS	
10:20–10:30	Break	
10:30–11:30	Defibrillation: AED introduction and 2-rescuer adult BLS	
11:30–12:30	Lunch	
12:30–12:40	Child BLS	
12:40–13:20	Infant BLS	
13:20–13:30	Adult/child choking, infant choking	
13:30–14:00	Written test	
14:00–14:40	Skill test – 1 and 2 rescuer adult BLS with AED skill test – 1 and 2 rescuer infant BLS skill test	
14:40–15:00	Wrap up	

**Course: difficult airway**		

**Time**	**First day**	
12:50–13:00	Registration	
13:00–13:10	Introduce faculty and participants	
13:10–13:30	Lecture	Introduction
13:30–14:10	Lecture	BMW and ETI with DL
14:10–14:50	Lecture	Difficult and failed airways
14:50–15:00	Break	
15:00–15:20	Lecture	Airway algorithms
15:20–15:50	Lecture	Video and optical laryngoscopy
15:50–16:20	Lecture	Capnography in airway management
16:20–16:40	Break	
16:40–17:10	Lecture	Demystifying the pediatric airway
17:10–17:40	Lecture	Medication-assisted intubation
17:40	Wrap up	

**Time**	**Second day**	
08:30–08:40	Registration	
08:40–10:40	Skill stations (rotate 4 times/30 min each)	
	A	Bag mask ventilation and direct laryngoscopy
	B	Extraglottic rescue devices
	C	Surgical airway rescue
	D	Endotracheal intubation *via* video/optical laryngoscopy
10:40–10:50	Break	
10:50–11:30	Interactive workshops (rotate 4 times/40 min each)	
	A	Pediatric cases
	B	Trauma cases
	C	Medical cases
	D	Unique EMS cases
11:30–12:30	Lunch	
12:30–14:30	A	Pediatric cases
	B	Trauma cases
	C	Medical cases
	D	Unique EMS cases
14:30–14:40	Break	
14:40–17:20	Test (rotate 4 times/40 min each)	
	A	Written test
	B	Trauma
	C	Medical
	D	Equipment practice
17:20–17:30	Final wrap up	

Halla-Stony EMEC also provided a difficult airway course for 2 days, including skill training with various airway devices to provide advanced simulation training focused on airway management and improve participants potential skill to life-threatening patients for pre-hospital emergency care as well as ACLS. Additionally, skill simulation practice was conducted using Q-CPR for 1 day with two American emergency medicine professors from Stony Brook university hospital in New York in October 2014. A Resusci Anne simulation course was held in October 2015 in Korea, and several BLS trial courses were managed by Vietnamese instructors at the advanced emergency education center of Hue UMP, since 2015. Therefore, basic skill training for two-way interactive techniques, cardiopulmonary skill report systems, and first airway courses, using many different types of airway management devices, were possible (20, 21). In addition to these courses, a problem-based learning/teaching method and repetitive teaching-and-learning small group practice were offered in Cheju Halla University. Surveys assessing satisfaction with the program were implemented immediately for each course and all the scores were over 85% in terms of course management, lecturer’s teaching method, time management, meals, and materials.

### Educational Infrastructure Development

Hue UMP had a vision to take the lead position in emergency medicine education in Vietnam, as they were the first to launch a resident program and symposium series in this developing area, based on both research and evidence (21). Therefore, advancement in the training environment, with regards to future simulation training capacity was considered. Essentially, the AHA’s list of equipment for international training organization approval was the priority for purchase.

The implementation of emergency medicine education center remodeling encountered some minor difficulties. Initially, the Hue UMP project team offered a separate space in the hospital for several labs, but this space did not fit requirements. However, a new building was under construction by way of funds from Atlantic Philanthropies in 2014. Therefore, the Korean project team drafted three design options for an education center, with expert consultation from a simulation equipment company and a construction company based in Vietnam. During the second invitational training, the Korean project team met twice with the Vietnam project team to determine their choice, and let them report to the Hue UMP Rector Board when they returned to Vietnam in December 2014 (15). The Vietnam project team reported to the Rector Board of Hue UMP and presented the blueprint of the simulation center. The Rector Board eventually approved use of a 355 m^2^ space on the fifth floor in the Hue learning resource center (20). The remodeling of emergency medicine education center was completed in March 2015 and an inauguration ceremony was held in September 2015.

The acquisition of the required lists of BLS training medical equipment and supplies were furnished to Hue UMP at the end of April 2015. Additionally, any equipment needed to build an appropriate simulation education center was also included. A teaching/learning system, centralized recording system for debriefing, Resusci Anne simulation equipment, a skill report system for calibration of chest compression and proper breathing volume, a simulation assist device (e.g., SimPad), a statistics package, and other airway management instruments as shown in Table 3 were included to purchase for research activities in the future (15).

**Table 3:** Furnished training equipment and remodeled laboratories.

List			Qty
Equipment	1	Little Anne (multi)	12
Education equipment	2	Baby Anne 4-Pack	3
Education equipment	3	LSR adult standard w/mask, in carton (IE)	12
Education equipment	4	LSR preterm complete w/masks, in carton (IE)	12
Education equipment	5	Pocket mask O2 HC (IE)	12
Education equipment	6	AED trainer (2)	12
Education equipment	7	CPR meter Europe—Region 1	1
Education equipment	8	Training mattress	12
Education equipment	9	Patient bed (3-motor)	4
Education equipment	10	Emergency stretcher	2
Education equipment	11	Dressing cart	4
Education equipment	12	SimPad system	1
Education equipment	13	Emergency cart	4
Education equipment	14	Resusci Anne QCPR full body trolley suit	1
Education equipment	15	SimPad skill reporter: International/English	1
Education equipment	16	Skill guide	1
Education equipment	17	Airways standard RA, Pkg of 24	1
Education equipment	18	Face skin decorated ×6	1
Education equipment	19	License key for skill reporter software for SimPad w/laptop	1
Education equipment	20	Little Jr. 4-pack	1
Education equipment	21	Pocket mask O2 HC (IE)	12
Education equipment	22	Laerdal pediatric PM, w/gloves in red/black SP	12
Education equipment	23	Standard training pads (1 set)	7
Remodeling	1	Basic life support (BLS) lab (interactive whiteboard), podium, desk and chairs (12 pairs), microphone, notebook (1 EA)	1
Remodeling	2	advanced cardiopulmonary life support lab (interactive whiteboard), podium, simulation recording system, microphone, notebook (2 EA)	2

### Mentoring Pilot BLS Training Course in Vietnam

Vietnamese BLS instructors initiated several pilot BLS courses for their medical and nursing students under the supervision of Korean BLS faculty, since 2015 at the BLS center in Hue UMP. However, Hue UMP was not a training site and only pilot trainings for adaptation of course management were implemented when the Korean project team visited Hue UMP.

Through the pilot experience, the project team could make a detailed future plan of coordination regarding how many BLS training sites need to be opened under the Hue UMP international training organization in Vietnam, how to provide training courses for medical and nursing students and licensed healthcare and non-healthcare personnel annually, and how to harmonize medical personnel supplementary training both in hospital and pre-hospital settings to provide sustainable training center maintenance before submitting the application for the international training organization to AHA.

### Approval as an International Training Organization by AHA

From the initiation of the first BLS provider course, October 2014 in Korea, it took 17 months to submit the international training organization application to AHA. Approval from the AHA to become an international training organization took approximately 6 months after submission. Fortunately, there was an international instructor refresher course in early March 2016 in Hong Kong. During this course, the Korean–Vietnam project team met the Hong Kong regional manager and three AHA representatives (director of international emergency cardiovascular care, resuscitation learning director, and a program development manager), and had the opportunity to discuss the launch of the international training organization and training center in Vietnam (22).

The AHA experts thought very highly of what had been achieved during the project, especially the aspects of quality infrastructure and personnel training. Additionally, a new international training organization approval was under consideration in the Asian region. However, most of applicants did not sufficiently meet AHA qualifications (15). Thus, the proposal put forth by the project team was very timely, and was therefore well positioned to obtain approval. The officer of international affairs agreed to begin the approval process, and planned to visit the facilities in Vietnam after completing several courses on the newly updated AHA guidelines. The future strategy on training was to use ebooks. Therefore, Internet infrastructure was a very important issue; however, the Vietnam facility exhibited appropriate information technology, which may have mirrored the AHA requirements.

## Discussion

The main indicator to evaluate the effectiveness of a national healthcare system is mortality rate of diseases and injuries. These statistics represent critical considerations for the improvement of a nation’s healthcare system to reduce national disease burden (23). Additionally, as recommended by the WHO, availability of quality healthcare personnel is an essential element to empower health systems. As such, personnel should be trained within an educational system that is appropriate for the needs of future healthcare prospects. However, the number of doctors and nurses per 1,000 citizens of Vietnam was below the recommended level of the WHO, and there were no paramedic or emergency medicine technicians in the pre-hospital stage. Vietnam’s total health expenditure was 6.9% of the gross domestic product in 2010 and equivalent to $85 USD per capita (16). Vietnam’s socioeconomic development plan toward 2020 explained that high-quality human resource development through comprehensive renovation of the national education system was one of three primary strategic methods for improvement (23).

The current project was entirely evaluated using five criteria of the Development Assistance Committee of the Organization for Economic Co-operation and Development. These included relevance, efficiency, effectiveness, impact, and sustainability (20, 24).

It was evaluated that the relevance of the current project was excellent in terms of reflecting Vietnam’s low level of development, but a high demand in the medicine education system area. Especially, this project was an ideal case in terms of the rapid advancement after the previous 1-year project supported by the Korea Foundation for International Healthcare, from 2013 to 2014 (12).

In the efficiency aspect, the Vietnam and Korea project teams needed to demonstrate a very close relationship to address the concerns of multiple stakeholders and obtain approval from the Vietnam government for implementation of the project, including tax-exemption of medical education equipment. Specifically, all the performance indicators were achieved within the project duration and the limited budget provided. In fact, the indicators of the establishment of an emergency medicine education system were accomplished in excess, as evidenced by the number of trained instructors, the furnishing of essential educational equipment, and the BLS training center appointment.

In the area of effectiveness, external evaluators were highly appreciative of the resource networking capacity of the Hue UMP building, and the open-mindedness to global partnerships on the part of Hue UMP to facilitate win–win strategies, rather than competition, in securing the training space, sharing the project results, and listening to feedback regarding the ongoing project (20). In addition, extra consultation in course management and equipment handling were provided thrice in Vietnam and twice in Korea to fulfill the metrics of instructor competence for international training organization approval.

With regards to impact, Hue UMP was expected to expand their training capacity to provide first-aid and BLS training for non-healthcare personnel. Located in central Vietnam, Hue UMP can begin to take on an international training organizational role to supervise all centers in Vietnam. The effects of project outcomes were witnessed as internal benchmarking in Vietnam. For example, a professor of Phu Tho medical college, located in the north of Hanoi, visited Hue UMP to identify advanced teaching and skill training methods, as well as sharing project outcomes in a seminar (20).

For sustainability, external evaluation experts reviewed that the outcome of the first phase of the project was attained as planned and also accomplished an unexpected positive effect (15, 20). It showed the importance of establishing a training system that can be operated by local experts, allowing for ownership, and guaranteeing the possibility of sustainable improvement. Previous research findings insist that an effective training system is the first step to building successful emergency medicine health systems (5–7). Hue UMP can now train BLS instructors, allowing for other BLS training sites to open, and train their medical, nursing, and other health allied students themselves.

Last, it should be mentioned that the selection criteria for invitation training was processed equally, regardless of gender or educational background in medicine or nursing. Even though doctors comprise more than 80% of the nursing faculty, potential future faculty candidates were considered with the same level of importance and priority during invitational training in Korea.

## Conclusion

The effects of constructing an education system can be judged by the percentage of project outcome indicators and the report of external experts’ evaluation, though the impacts of the project cannot be judged by short-term results. The indicators of project performance exceeded 100% as shown in Table 4 and received an excellent result in all OECD-DAC evaluation criteria.

**Table 4:** Summary of performance indicators and the outcome.

Goal	Objectives	Activities	Indicator	Outcome	Result
Increase survival rate of people with life-threatening conditions	Construct emergency medicine education system	Providing basic life support (BLS) course	3 BLS instructors per year	9 instructors over 2 years	150% achievement
		Mentoring pilot course management	Two times per year	4 times	100% achievement
		Renovate infrastructure	Renovation within first year	Remodeled BLS center in 2015	100% achievement
		Furnish appropriate training equipment	Provision of mandatory and optional equipment to be approved by American Heart Association (AHA)	All the equipment were provided in 2015	100% achievement
		Readiness for approval of AHA BLS training site	All the requirement met within project year	Received promise to approval in March, 2016	Early achievement

In a national healthcare system, personnel training with foresight into future demand are necessary to improve the responsiveness of the system and the overall health of the population (4, 16). Vietnam is a country that has demonstrated dramatic healthcare system growth, in accordance with socioeconomic development. Through this first international emergency medicine focused on BLS and ACLS training center in Vietnam, Hue UMP has proven its geopolitical position as the primary provider of high-quality health services to 25 million inhabitants of the central region of Vietnam, and as a coordinating training site in North and South regions of Vietnam. Further, Hue UMP can also take the role of coordinating with and training healthcare personnel from Laos and Cambodia, in addition to the approximately 100,000 healthcare personnel from 63 provinces nationally. Hue UMP’s vision for the 5 years (2016–2020) is to train approximately 1,000–2,000 students and 500 health staff in the first year, and it seeks to increase the total number of those trained to more than 10,000 per year by 2020. During this time period, in addition to the current BLS provider courses, we would also like to conduct BLS instructor courses, ACLS courses, and PALS courses. Similar to the experience of Korea in increasing the in-hospital survival rate from 17.2% in 2005 to 28.5% in 2009 (25), increasing the rate of bystander chest compression from 7.78% to 20.13% between 2005 and 2009 to 2010 and 2014 (26), and the addition of qualified heart savers by paramedics and analysis of resuscitation training effects (27, 28), Vietnam’s indicators on this issue will also likely change in the future.

The question then arises as to what is needed for a sustainable outcome. To adequately examine this, there are several issues to be considered. First, policy formation and regulations related to healthcare personnel training are in need of amendment to support the expansion of international standard skills, and teamwork training, in an effort to increase efficiency and improve the overall health of the population. Recently, health departments in Vietnam regulated mandatory supplemental training to healthcare personnel. As such, if specific training of BLS and ACLS training were to be required by health authorities, this would likely exert a positive impact on the quality improvement of healthcare personnel. Additionally, the 115 pre-hospital services in each region should also require this type of formal and standardized training for their staff. Second, injuries by accidents frequently occur away from hospitals. Therefore, it is necessary to build community capacity in emergency first-aid. One barrier to this may be that there is no legal permission to allow the use of automated electric defibrillators by non-healthcare personnel in Vietnam. However, this issue can be resolved by the training of people in the community, people with qualified training personnel, and an infrastructure based on legal protection.

## Author Contributions

All authors listed, have made substantial, direct and intellectual contribution to the work, and approved it for publication.

## Conflict of Interest Statement

The authors declare that the research was conducted in the absence of any commercial or financial relationships that could be construed as a potential conflict of interest.
